# The role of early life factors in the development of ethnic differences in growth and overweight in preschool children: a prospective birth cohort

**DOI:** 10.1186/1471-2458-14-722

**Published:** 2014-07-15

**Authors:** Lenie van Rossem, Esther Hafkamp-de Groen, Vincent WV Jaddoe, Albert Hofman, Johan P Mackenbach, Hein Raat

**Affiliations:** 1 The Generation R Study Group, Erasmus MC-University Medical Centre Rotterdam, Dr. Molewaterplein 50, Rotterdam 3015 GE, the Netherlands; 2 Department of Public Health, Erasmus MC-University Medical Centre Rotterdam, Dr. Molewaterplein 50, Rotterdam 3015 GE, the Netherlands; 3 Julius Center for Health Sciences and Primary Care, University Medical Center Utrecht, PO Box 85500, Utrecht 3508 GA, the Netherlands; 4 Department of Paediatrics, Erasmus MC-University Medical Centre Rotterdam, Dr. Molewaterplein 50, Rotterdam 3015 GE, the Netherlands; 5 Department of Epidemiology, Erasmus MC-University Medical Centre Rotterdam, Dr. Molewaterplein 50, Rotterdam 3015 GE, the Netherlands

**Keywords:** Ethnicity, Preschool, Overweight, Epidemiology, Infant weight gain, Socio-economic status, Breastfeeding, BMI

## Abstract

**Background:**

Ethnic differences in childhood and adulthood are known, but ethnic differences in preschool overweight and associated factors are less studied. We assessed ethnic differences in pre-school age overweight, and studied the mediating role of early life factors in this association. Furthermore, we assessed body mass index (BMI) z-score development from birth to age 4 years to study ethnic-specific differences in BMI z-score trajectory.

**Methods:**

We used data on 4581 children participating in a birth cohort who were born between 2002 and 2006 in Rotterdam, the Netherlands. Child’s ethnicity was defined according to country of birth of the parents. Weight and length/height was repeatedly measured between 1 and 45 months of age. Overweight at age 4 years was defined according to cut-off points for BMI from the international obesity task force. We performed logistic regression to obtain independent estimates of the association between ethnicity and preschool-age overweight, and to assess the mediating role of early life risk factors. Mixed models were used to describe BMI-z development for each ethnic group from birth to preschool age.

**Results:**

Relative to native Dutch children, non-Dutch children were more likely to be overweight at age 4 years, except for Surinamese-Hindustani children. Socio-demographic factors, parental BMI, and infant weight change in the first 6 months after birth reduced associations. After full adjustment, Turkish (OR: 2.02, 95% CI: 1.34-3.04) and Antillean/Surinamese Creole (OR: 1.78, 95% CI: 1.06-3.02) children were still more likely to be overweight at age 4 years.

**Conclusion:**

Ethnic differences on the prevalence of overweight in preschool children can be partially explained by maternal educational level, parental overweight and early infant weight change. These may be possible targets to reduce ethnic inequalities in preschool age overweight.

## Background

Throughout the developed world, childhood overweight is increased in ethnic minority groups. In the USA, childhood overweight prevalence is higher among Blacks and Hispanics than among Caucasians [1]; and in the UK, overweight prevalence is higher among South-Asian and African-Caribbean children, although inconsistencies arise when different definitions of overweight are used [2]. Studies from Australia, Germany and the Netherlands also report that certain ethnic groups have a higher prevalence of childhood overweight [3-5]. It is important to elucidate these ethnic differences in childhood overweight in order to take measures to prevent overweight in these groups. Ethnic differences in overweight have been reported to be present from preschool age onwards [3,5,6]. This suggests that factors responsible for ethnic differences in overweight act in early life. Indeed, one study reported ethnic differences in several risk factors (including infant feeding, sugar-sweetened beverages, and mother's feeding practices) in preschool children, but this study did not assess the contribution of these risk factors to ethnic inequalities in overweight [7].

We assessed ethnic differences in pre-school age overweight, and studied the mediating role of risk factors that were present before overweight was developed (‘early life factors’) in this association. We distinguished these early life factors into parental, prenatal, and postnatal factors in order to get clues which of these factors can be targets for preventive interventions. Furthermore, we assessed body mass index (BMI) z-score development from birth to age 4 years to study ethnic-specific differences in BMI z-score trajectory.

## Methods

### Study design

This study was embedded in Generation R, a population-based multi-ethnic prospective cohort study from fetal life onwards. Invitations to participate in the study were made to all pregnant mothers who had an expected delivery date between 2002 and 2006 and who lived in the study area (Rotterdam, the Netherlands). Details of the study are described elsewhere [8].

The study was conducted in accordance with the guidelines proposed in the World Medical Association Declaration of Helsinki, and was approved by the Medical Ethical Committee at Erasmus MC, University Medical Centre Rotterdam. Written consent was obtained from all participants.

### Study population

Consent for postnatal follow-up was available for 7893 children. We excluded twins from the analyses (*n* = 197). To avoid clustering, our analyses excluded data on the second or third pregnancy of any woman who was participating in the Generation R study with more than one child (*n* = 580). We excluded participants of whom we missed ethnicity (*n* = 657). Children who had less than 4 measures on height or weight were excluded (*n* = 734), as they were often lacking data on later overweight. In total, data of 5725 subjects were available for analyses. Children with a European (*n* = 466), Indonesian (*n* = 76), Asian western (*n* = 5), Asian non-Western (*n* = 161), American Western (*n* = 30), American non-Western (*n* = 84), Oceania (*n* = 10), African (*n* = 118) and Surinamese-other (*n* = 194) ethnicity were excluded from the analyses because of their small sample size and heterogeneity (*n* = 1144). After exclusion of these, the study sample consisted of 4581 children.

### Measurements

#### Child’s ethnicity

We used the standard definition of Statistics Netherlands as a basis for defining ethnic background. This definition is based on country of birth of the parents [9], which we extended to country of birth of parents and grandparents. A child was considered as having an immigrant background if one of both parents was born abroad. If both parents were born abroad, but in different countries, mother’s country of birth was leading. We also take third generation immigrants into account: if both parents were born in the Netherlands, but at least two grandparents of the same parent were born abroad, the child was assigned ethnicity of these grandparents. Also, children with one parent born abroad, but four grandparents born in the Netherlands, were assigned a Dutch ethnicity. All possible combinations and their consequences for assignment of child’s ethnicity are described in Additional file 1.

We initially distinguished six ethnic groups according to country of birth. Surinamese children were further subdivided according to self-assigned ethnicity of the mother because different ethnicities live in that country. We performed analyses on 3126 native Dutch, 474 Turkish, 351 Moroccan, 199 Cape Verdean, 295 Antillean/Surinamese-Creole, and 136 Surinamese-Hindustani children (*n* = 4581).

#### BMI and overweight

Length (to 24 months)/height (from 24 months) and weight were measured according to a standard schedule and procedures by a well-trained staff at each visit to the child health centre. Length was measured in supine position to the nearest millimetre until the age of 24 months using a neonatometer, after which height was measured in standing position by a Harpenden stadiometer (Holtain Limited, Dyfed, U.K.). Weight was measured using a mechanical personal scale to the nearest 0.01 kg (SECA, Hamburg, Germany). A child usually visits the child health center that is assigned according to postal code. Routine visits at the child health centers are scheduled around 1, 2, 3, 4, 6, 11, 14, 18, 24, 30, 36, and 45 months of age. BMI was calculated as weight/height^2^. BMI-for-age z-scores were derived from national growth curves [10]. We used the age and gender specific cut-off points of the International Obesity Task Force to define overweight [11].

#### Covariates

##### Socio-demographic variables

All socio-demographic variables were assessed by questionnaire at study enrollment. Household income was dichotomized (< € 1600 per month, ≥ € 1600 per month). Households that earn below this cut-off point are considered as low-income groups [12]. Level of maternal education was established at enrollment [13]. The mothers were classified into three educational categories according to the highest level of education they had attained: low (no education; primary school; lower vocational training; intermediate general school; or three years general secondary school); middle (>3 years general secondary school; intermediate vocational training; or first year of higher vocational training), and high (higher vocational training; or Bachelor’s degree, higher academic education). The following question was asked to determine material hardship: ‘Do you have any difficulty in paying food, rent, electricity bill and such like?’ Answer categories included ‘no’ (no), ‘some’ (yes), and ‘great’ (yes) difficulties. Single motherhood (yes, no) was obtained by questionnaire.

##### Parental characteristics

Mother’s BMI was calculated from self-reported pre-pregnancy weight and measured height at enrollment. Father’s BMI was calculated from measured weight and height at enrollment. Smoking during pregnancy (yes, no) was derived from the three prenatal questionnaires (1^st^, 2^nd^, and 3^rd^ trimester).

##### Birth characteristics

Birth weight (grams) and gestational age (weeks) were obtained from medical records.

##### Postnatal factors

Information on breastfeeding was obtained by a combination of questionnaires administered at 2, 6 and 12 months after birth. Questions included items on the proportion of breastfeeding relative to infant formula, breastfeeding duration, and number of daily feedings. Breastfeeding was defined as having received any (including non-exclusive) breastfeeding for 6 months. Infant weight change was defined as the difference in BMI between birth and six months of age.

### Statistical analyses

We assessed characteristics of participants for the total population and for each ethnicity separately. P-values for differences in risk factors for overweight between ethnic groups were calculated by means of the Chi-square test for categorical variables and ANOVA for continuous variables.

First, we performed logistic regression to obtain independent estimates of the association between ethnicity (reference: 'Dutch') and preschool overweight, and to assess the mediating role of early life risk factors. We built our models in the following steps. First, we assessed the association between ethnicity and childhood overweight, adjusted for age and sex (model 1). Then, we adjusted separately for the socio-demographic variables: household income, mother’s educational level, and material hardship (model 2), other parental variables: parental BMI and maternal smoking during pregnancy (model 3), birth characteristics: birth weight and gestational age (model 4), and postnatal risk factors: breastfeeding and infant weight gain (model 5) to show the mediating role of each of these groups on the association between ethnicity and overweight. Lastly, we adjusted for all variables simultaneously (model 6).

To prevent bias, loss of information and enhance comparison between models, we applied multiple imputation in SPSS v17.0 on the early life risk factors to this analysis [14,15]. The missing values for the covariates ranged from 0% (birth weight, gestational age) to 40% (infant weight gain). The number of missing values were higher for non-Dutch participants for the variables father’s BMI, household income, material hardship and breastfeeding. We assumed the missings to be random (MAR). Five imputed datasets and their pooled estimates were generated using a fully conditional specified model to handle missing values. Imputations were based on the relations between all covariates in the study. We used the pooled estimates from these five imputed datasets to report odds ratios (OR) and their 95% confidence intervals (CI).

Second, repeated measurements analysis ('PROC MIXED' procedure in SAS) was performed to calculate whether BMI z-score curves differ per ethnicity throughout the first 4 years of life. This analysis takes into account that data are correlated within one individual. The best fitting model for BMI as a function of age was built using fractional polynomials. The best fitting model for BMI z-score was BMIZ = ß_0_ + ß_1_*(ln)age + ß_2_*√age. To this model we added ethnicity as a main determinant (reference: Dutch), and an interaction term of ethnicity with both transformations of age. We modeled the BMI z-score development of each ethnic group for the total population and stratified for overweight and normal weight children. The best model for the subsamples was: BMIZ = ß_0_ + ß_1_*age + ß_2_*√age.

Analyses were conducted with the Statistical Package for Social Sciences (SPSS) version 17.0 for Windows (SPSS Inc, Chicago, IL, USA). Statistical Analysis Software (SAS), version 9.1.3 for Windows (SAS Institute, Cary, NC, US) was used for the repeated measurements analyses (mixed procedure).

## Results

### Sample characteristics

Ethnic differences were present in all covariates (Table 1). Generally, non-Dutch mothers were lower educated, had a lower household income, suffered more often from material hardship, and were more often single (p < 0.001) compared to native Dutch mothers. The direction of differences in father’s BMI, smoking during pregnancy, breastfeeding, birth weight and gestational age varied according to ethnic subgroup (Table 1). Prevalence of overweight between 2 and 4 years was about 10%. Relative to Dutch children whose overweight prevalence varied between 10 and 12% between ages 2 to 4, overweight prevalence was consistently higher in Moroccan (14-21%, p < 0.001) and Turkish (21-28%, p < 0.001) children, and lower in Surinamese-Hindustani children (0-8%, p < 0.001) (Table 1).

**Table 1:** Subject characteristics for 4581 children of different ethnicities in the Generation R Study

		**Total (n = 4581)**	**Dutch (n = 3126)**	**Turkish (n = 474)**	**Moroccan (n = 351)**	**Cape Verdean (n = 199)**	**Antillean/Surinamese Creole (n = 295)**	**Surinamese-Hindustani (n = 136)**	**p-value**^**a**^
**Socio-demographic variables**									
Mothers education	% low	9.0	2.6	32.8	24.2	21.8	12.5	14.1	<0.001
	% middle	43.3	34.7	53.7	62.1	66.3	70.6	71.9	
	% high	17.7	62.7	13.5	13.7	11.9	17.0	14.1	
Household income	% low	24.1	8.6	63.5	67.9	64.6	67.0	51.5	<0.001
Material hardship	% yes	18.1	9.2	46.0	36.8	41.1	45.3	32.0	<0.001
Single motherhood	% yes	11.2	5.3	6.7	8.9	48.2	53.2	23.0	<0.001
**Other parental characteristics**									
BMI mother	In kg/m^2^	23.7 (4.3)	23.1 (3.8)	25.2 (5.1)	25.3 (4.6)	23.8 (3.9)	24.6 (5.2)	23.5 (4.7)	<0.001
BMI father	In kg/m^2^	25.2 (3.5)	25.1 (3.3)	26.3 (4.0)	25.8 (3.8)	24.5 (3.1)	25.3 (3.9)	24.5 (3.6)	<0.001
Pregnancy smoking	% yes	11.0	10.4	16.0	8.0	11.2	12.2	10.3	0.01
**Birth characteristics**									
Birth weight	In grams	3449 (550)	3500 (553)	3393 (492)	3518 (471)	3271 (531)	3221 (558)	3041 (516)	<0.001
Gestational age	In weeks	40.0 (1.7)	40.0 (1.7)	39.9 (1.6)	40.3 (1.5)	39.9 (1.4)	39.6 (1.8)	39.4 (1.5)	<0.001
**Risk factors in infancy**									
Breastfeeding at 6 months	% yes	28.8	29.6	37.3	25.9	18.9	18.2	17.0	<0.001
**Child overweight**	Age 2	11.3	9.2	21.4	20.7	13.4	12.6	3.0	<0.001
	Age 2.5	11.4	8.4	26.3	17.3	20.8	10.2	0.0	<0.001
	Age 3	10.1	7.8	23.3	17.9	9.7	11.7	1.2	<0.001
	Age 3.5	10.4	7.6	28.1	14.0	12.5	10.0	4.5	<0.001
	Age 4	12.1	9.2	23.1	18.9	17.4	16.1	8.0	<0.001

Values are percentages or mean (SD).

^a^P-values are for Chi^2^ test for categorical variables, and ANOVA for continuous variables.

Missing values were 103 (2.2%) for educational level, 940 (20.5%) for income, 988 (21.6%) for material hardship, 82 (1.8%) for single motherhood, 943 (20.6%) for BMI mother, 1229 (26.8%) for BMI father, 813 (17.7%) for smoking during pregnancy, 2 (0%) for birth weight, 719 (15.7%) for breastfeeding at 6 months.

### Contribution of risk factors in explaining ethnic overweight differences

Of the 4581 children in the study population, 2994 (65%) had measured weight and height at the last scheduled visit in the child health centers (around 45 months of age). Unadjusted analyses showed that all non-Dutch groups, except the Surinamese-Hindustani children, were more likely to be overweight at age 4 (Table 2, model 1). Adjustment for mother's educational level (model 2), parental BMI (model 3), and infant weight change (model 5) attenuated the associations, with the strongest attenuation after adjusting for mother's educational level. In contrast, perinatal factors strengthened associations (model 4). After full adjustment (model 6), only Turkish (OR: 2.02, 95% CI: 1.34-3.04) and Antillean/Surinamese-Creole (OR: 1.78, 95% CI: 1.06-3.02) ethnicity were independently associated with overweight at age 4. Associations between Moroccan and Cape Verdean ethnicity and preschool overweight at age 4 were not statistically significant after full adjustment for early life risk factors.

**Table 2:** **Associations between ethnicity and overweight at 48 months, and contribution of early life factors in the Generation R Study (n = 2994)**^**a**^

	**Model 1**	**Model 2**	**Model 3**	**Model 4**	**Model 5**	**Model 6**
Child’s ethnicity						
*Native Dutch*	1 (ref)	1 (ref)	1 (ref)	1 (ref)	1 (ref)	1 (ref)
*Turkish*	**3.25 (2.39-4.41)**	**2.14 (1.47-3.11)**	**2.62 (1.90-3.63)**	**3.69 (2.69-5.06)**	**3.04 (2.23-4.15)**	**2.02 (1.34-3.04)**
*Moroccan*	**2.40 (1.66-3.47)**	**1.65 (1.08-2.52)**	**1.88 (1.28-2.78)**	**2.63 (1.81-3.83)**	**2.31 (1.59-3.36)**	1.48 (0.92-2.38)
*Cape Verdean*	**2.00 (1.22-3.27)**	1.39 (0.81-2.38)	**1.94 (1.15-3.25)**	**2.63 (1.59-4.37)**	**1.88 (1.14-3.11)**	1.78 (0.99-3.20)
*Antillean/Surinamese-Creole*	**1.82 (1.16-2.87)**	1.46 (0.89-2.40)	**1.63 (1.01-2.62)**	**2.25 (1.41-3.57)**	**1.85 (1.17-2.92)**	**1.78 (1.06-3.02)**
*Surinamese-Hindu*	0.89 (0.41-1.96)	0.68 (0.30-1.54)	0.81 (0.36-1.83)	1.39 (0.62-3.09)	0.88 (0.49-1.94)	1.04 (0.44-2.47)
Household income						
*Low*		0.93 (0.62-1.38)				1.03 (0.70-1.53)
*Above average*		1 (ref)				1 (ref)
Mother’s educational level						
*High*		**0.51 (0.36-0.73)**				**0.61 (0.41-0.89)**
*Middle*		**0.35 (0.23-0.54)**				**0.48 (0.30-0.77)**
*Low*		1 (ref)				1 (ref)
Material hardship						
*No*		1 (ref)				1 (ref)
*Yes*		1.19 (0.97-1.80)				1.13 (0.75-1.71)
Mother’s BMI (per unit)			**1.07 (1.04-1.10)**			**1.05 (1.02-1.08)**
Father’s BMI (per unit)			**1.15 (1.09-1.20)**			**1.14 (1.08-1.21)**
Smoking during pregnancy						
*No*			1 (ref)			1 (ref)
*Yes*			1.36 (0.85-2.18)			1.49 (0.96-2.31)
Birth weight (per 500 gram)				**1.05 (1.04-1.07)**		**1.06 (1.04-1.07)**
Gestational age (per week)				**0.85 (0.78-0.93)**		**0.88 (0.80-0.96)**
Breastfeeding at 6 months						
*No*					1 (ref)	1 (ref)
*Yes*					1.26 (0.98-1.62)	**1.33 (1.02-1.75)**
Difference in BMI between birth and 6 months (per unit)					**1.29 (1.17-1.42)**	**1.41 (1.26-1.58)**

^a^Values are odds ratios (95% confidence intervals). Values in bold indicate statistical significance.

Model 1: adjusted for age and sex.

Model 2: adjusted for sex, age, and socio-demographics (household income, mother's education, material hardship).

Model 3: adjusted for sex, age, and parental (parental BMI, maternal smoking during pregnancy).

Model 4: adjusted for sex, age, and birth characteristics (birth weight and gestational age).

Model 5: adjusted for sex, age, and postnatal factors (breastfeeding, infant weight gain).

Model 6: adjusted for all variables in model 1–5 simultaneously.

The analysis based on the imputed and not imputed data revealed the same early life overweight risk factors for model 1–5 (see Additional file 2). For the final model (model 6), the risk factors for overweight slightly differed. Both methods revealed paternal BMI, birth weight, and infant weight change as risk factors. In the imputed data, educational level, mother’s BMI, gestational age, and breastfeeding were risk factors for childhood overweight. Although the effect estimates pointed in similar directions, these factors were not statistically significant.

The association between ethnicity and overweight followed a similar pattern for the not imputed data; after adjustment for the socio-demographic, parental, and postnatal factors the association between ethnicity and overweight was attenuated, while adjustment for the perinatal factors strengthened the association.

### BMI development in ethnic groups

**Figure 1:**
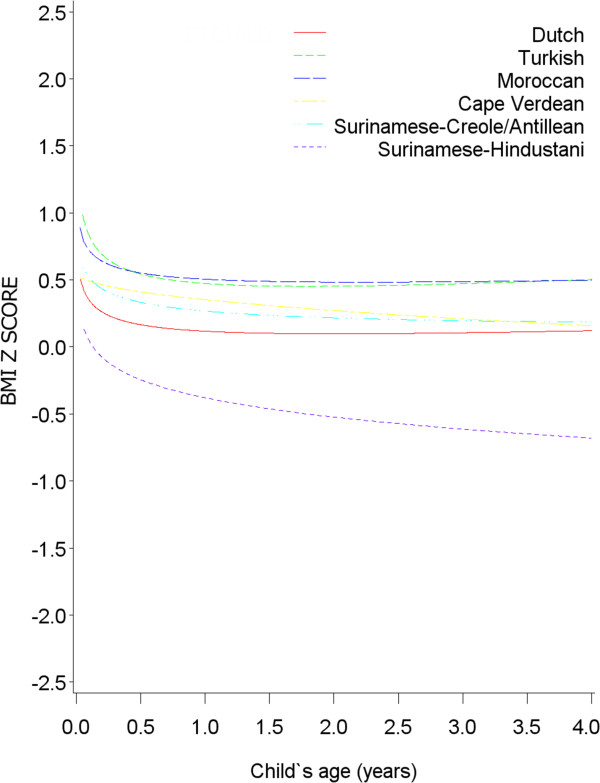
**BMI development (in z-score) until age 4 for each ethnic group (n = 4581, obs = 36904).** Equation: BMI z score = -0.13 + 0.18*Turkish + 0.42*Moroccan + 0.67*Cape Verdean + 0.28*Surinamese-Creole -0.18*Surinamese-Hindustani +0.24*√age*Dutch +0.41*√age*Turkish +0.21 *√age*Moroccan -0.19*√age*Cape Verdean + 0.12*√age*Surinamese-Creole -0.08*√age*Surinamese-Hindustani -0.17*ln(age)*Dutch -0.28*ln(age)*Turkish -0.16*ln(age)*Moroccan -0.003*ln(age)*Cape Verdean -0.14*ln(age)*Surinamese-Creole -0.16*ln(age)*Surinamese-Hindustani.

**Figure 2:**
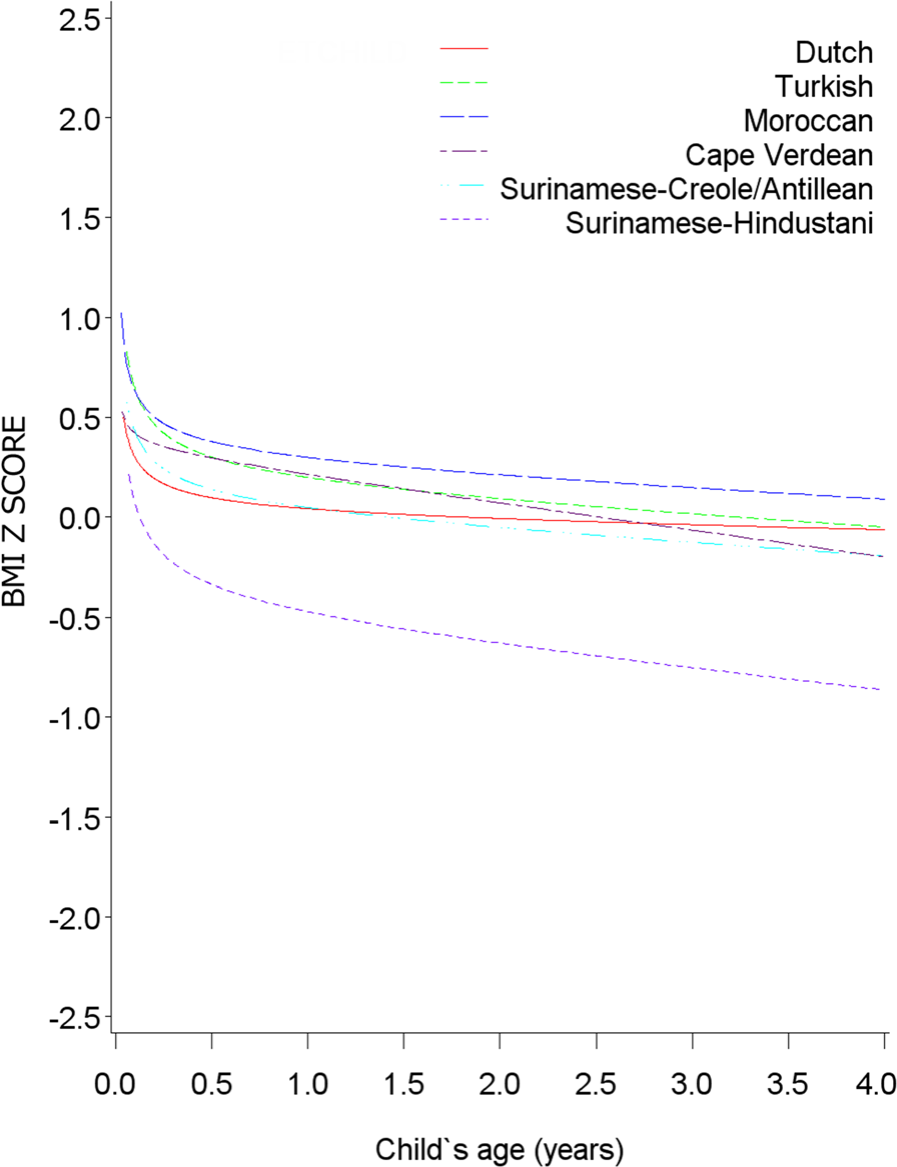
**BMI development (in z-score) per ethnic group until age 4 in normal-weight children (n = 2643, obs = 22806).** Equation: BMI z score = -0.59 + 0.53*Turkish + 0.65*Moroccan + 0.80*Cape Verdean + 0.89*Surinamese-Creole +0.54*Surinamese-Hindustani +0.11*√age*Dutch +0.18*√age*Turkish +0.13 *√age*Moroccan +0.04*√age*Cape Verdean + 0.15*√age*Surinamese-Creole +0.21*√age*Surinamese-Hindustani -0.02*age*Dutch -0.05*age*Turkish -0.05*age*Moroccan -0.13*age*Cape Verdean -0.05*age*Surinamese-Creole -0.10*age*Surinamese-Hindustani.

**Figure 3:**
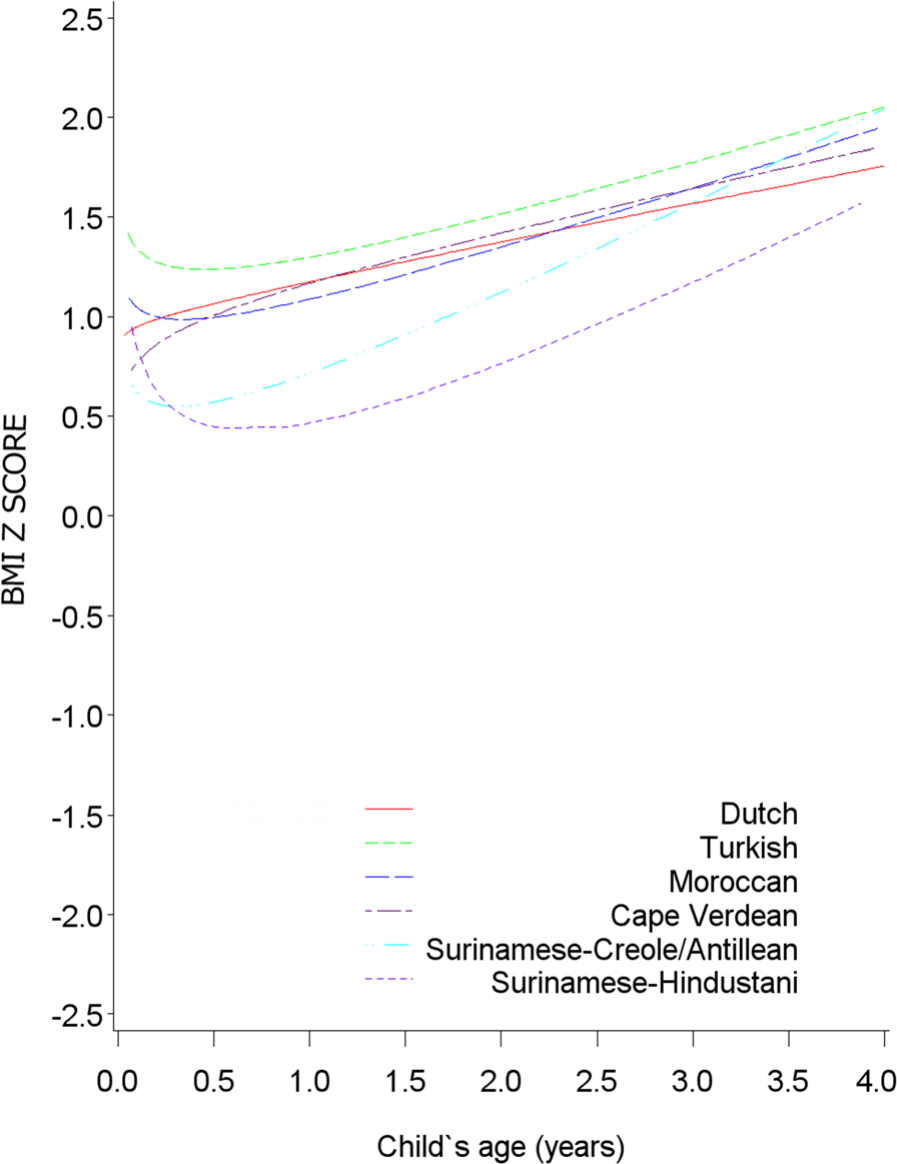
**BMI development (in z-score) per ethnic group until age 4 in overweight children (n = 351, obs = 2940).** Equation: BMI z score = -0.10 + 1.09*Turkish + 1.08*Moroccan + 0.84*Cape Verdean + 1.08*Surinamese-Creole +0.30*Surinamese-Hindustani +0.03*√age*Dutch -0.14*√age*Turkish -0.12 *√age*Moroccan +0.10*√age*Cape Verdean -0.16*√age*Surinamese-Creole -0.38*√age*Surinamese-Hindustani +0.18*age*Dutch -0.32*age*Turkish +0.34*age*Moroccan +0.18*age*Cape Verdean +0.52*age*Surinamese-Creole +0.56*age*Surinamese-Hindustani.

We assessed BMI development without any of the explanatory variables in the model to study specific time periods in which BMI differences between ethnic groups could occur. The BMI development model indicated differences in BMI development between ethnic subgroups (p-value for ethnicity: 0.007, p-value for ethnicity*ln(age): <0.0001, p-value for ethnicity*√age: <0.0001). All non-Dutch ethnic groups except for Surinamese-Hindustani children, had a higher BMI z-score from 1 month of age onwards (Figure 1). Development of BMI z-score was similar for all ethnic groups, as indicated. This was also the case when restricting the sample to normal weight children (Figure 2), but BMI z-score was lower in Cape Verdean and Antillean/Surinamese-Creole children at the time preschool age was reached. Also, Antillean/Surinamese-Creole overweight children had a steeper increase in BMI z-score in early life than the other ethnic groups (Figure 3).

## Discussion

### Main findings

This study shows that relative to native Dutch children, non-Dutch children are more likely to develop overweight at a very young age, with the exception of Surinamese-Hindustani children. BMI differences are already present in early postnatal life, and develop in a persistent way to preschool age. Mother's educational level, higher parental BMI, and a larger infant BMI change between birth and 6 months of age were important contributors in explaining the higher prevalence of overweight in non-Dutch children.

### Interpretation

The results of our study are consistent with the findings of a study that reported a higher prevalence of overweight in Turkish and Moroccan children from the age of 3 years onwards [3,6]. Similar findings were also reported in Australia for Mediterranean white children from the age of 5 years onwards [5]. In contrast to the results of Whitaker and Orzol, who concluded that the higher prevalence of overweight in Hispanic children could not be explained by maternal education, household income or food security, our study showed a large contribution of maternal educational level [16]. This may be due to the difference in country-specific ethnicities, and underlying history of migration. In our study, parental BMI and infant weight change also explained part of the association between ethnicity and childhood overweight. Another Dutch birth cohort study reported similar contributing factors [17]. Rapid infant weight gain is a well-established risk factor for later overweight [18]. Gain in infant BMI between birth and 6 months might be driven by infant feeding practices. Woo et al. reported that breastfeeding (comparing those receiving any breastfeeding for 4 months versus those receiving any breastfeeding for less than 4 months) mediates the association between ethnicity and overweight in adolescents [19]. Although breastfeeding was a strong determinant of overweight in our study, we did not see a mediating effect of breastfeeding on the association between ethnicity and overweight until age 4 years. The protective effect of breastfeeding on overweight may express at a later age. Also, we did not study complementary feeding. It has been reported that some ethnic groups do not adhere to the feeding recommendations, which may result in overfeeding [20,21]. Cultural differences may play an important role in overfeeding, as a ‘chubby’ baby is often seen as the most healthy baby in some ethnic groups [22]. Thus, part of the association may be due to feeding practices other than breastfeeding that is reflected in faster infant weight gain. However, determinants of infant weight gain can also be genetic or based on developmental factors during pregnancy [23,24].

### Study limitations and strengths

Strengths of this study were the longitudinal design with repeated measurements of BMI and the homogeneous ethnic groups. However, ethnicity is not a standardized, well-defined concept. It refers to people belonging to the same nation, religion, language, country of birth, or culture [25]. We used country of birth because it is the most objective and stable measure that can be used in young children. Nevertheless, country of birth does not cover all aspects of ethnicity, such as culture and ethnic identity [26]. Also, we categorized the children of mixed ethnicity according to ethnic background of the mother’s family, which may have attenuated our results. Future studies could give the associations between several aspects of ethnicity and overweight.

We used BMI as an indicator of overweight and obesity. BMI is a measure of excess weight rather than of excess fat. Fat percentage may differ for BMI levels among ethnic subgroups: this is especially the case in Asians [2]. However, there are no specific cut-off points available for Asians to define overweight and obesity according to the BMI [27]. DXA measures for this cohort will become available in the future.

Lastly, the percentages of mothers from ethnic minorities are lower among the participants than expected from the population data in Rotterdam [28]. This could have led to selection bias if associations between ethnicity and overweight are different for those who participate compared to those who do not participate. Although this is unlikely and associations are probably unaffected, overweight prevalence may not be generalized to the ethnic minority population. Also, a substantial number of values in the covariates were missing, and non-Dutch participants had a higher number of missing values for some of the covariates; however, on the assumption that these are missing at random (i.e. missings may be correlated with variables in the model, but may not be correlated with variables not in the model) they are unlikely to have caused selection bias. We assume missing at random because associations between covariates and overweight were in expected directions, as reported in earlier studies. Also, associations between ethnicity and overweight were in similar direction as a previous Dutch study [17]. In addition, despite the loss of power in the not imputed data, the interpretation of the imputed and not imputed data was similar.

## Conclusion

This study shows that ethnic inequalities in preschool-age overweight have their origins in factors that operate before (maternal educational level), during (parental BMI), and after (infant weight change) pregnancy. After birth, BMI development was fairly similar between ethnic groups. Maternal educational level, parental BMI, and infant weight change explained part of the ethnic differences in childhood overweight. Therefore, in order to reduce ethnic inequalities in childhood overweight, prevention programs should start in the earliest stage of pregnancy. Future research should clarify the underlying behavioral factors of ‘maternal education’ , ‘parental BMI’ , and ‘infant weight change’.

## Abbreviations

OR: Odds ratio; AOR: Adjusted odds ratio; CI: Confidence interval; BMI: Body mass index.

## Competing interests

The authors have declared that no competing interests exist.

## Authors’ contributions

LvR conceptualized the study, analyzed the data, and drafted the manuscript. EH participated in the concept and design of the study, assisted in analyzing and drafting the manuscript, and helped with interpretation of the results. VWJ and AH were involved in data acquisition, participated in concept and design of the study, and critically revised the manuscript. JPM was involved in data acquisition, supervised the study, participated in concept and design of the study, and critically revised the manuscript for important intellectual content. HR had the idea for the study, supervised the study, participated in concept and design, interpreting the data and revised several drafts of the manuscript. All authors have read and approved the submission of this version of the manuscript.

## Pre-publication history

The pre-publication history for this paper can be accessed here:

http://www.biomedcentral.com/1471-2458/14/722/prepub

## Supplementary Material

Additional file 1 Definition of ethnicity. Click here for file

Additional file 2 **Main results (Table** 2**) for unimputed data (available case analysis).** Click here for file
