# Supplementary material for: The role of early life factors in the development of ethnic differences in growth and overweight in preschool children: a prospective birth cohort
Source: BMC Public Health. 2014 Jul 15;14:722. doi: 10.1186/1471-2458-14-722 (PMC4227130; doi:10.1186/1471-2458-14-722)
Supplement: Additional file 1 — Definition of ethnicity. [file 1471-2458-14-722-S1.doc]

Appendix 1: Assignment of child’s ethnicity according to country of birth parents and grandparents

| Child’s ethnicity | Country of birth of: | | | | | |
| --- | --- | --- | --- | --- | --- | --- |
|  | Mother | Father | Mother’s mother (grandmother) | Mother’s father  (grandfather) | Father’s mother (grandmother) | Father’s father (grandfather) |
| ***Dutch*** | Netherlands | Netherlands | Netherlands | Netherlands | Netherlands | Netherlands |
|  | Netherlands | Netherlands | Abroad | Netherlands | Netherlands | Netherlands |
|  | Netherlands | Netherlands | Netherlands | Abroad | Netherlands | Netherlands |
|  | Netherlands | Netherlands | Netherlands | Netherlands | Abroad | Netherlands |
|  | Netherlands | Netherlands | Netherlands | Netherlands | Netherlands | Abroad |
|  | Netherlands | Netherlands | Abroad | Netherlands | Abroad | Netherlands |
|  | Netherlands | Netherlands | Abroad | Netherlands | Netherlands | Abroad |
|  | Netherlands | Netherlands | Netherlands | Abroad | Abroad | Netherlands |
|  | Netherlands | Netherlands | Netherlands | Abroad | Netherlands | Abroad |
|  | Netherlands | Abroad | Netherlands | Netherlands | Netherlands | Netherlands |
|  | Abroad | Netherlands | Netherlands | Netherlands | Netherlands | Netherlands |
| ***non-Dutch*** |  |  |  |  |  |  |
| *second generation* | Netherlands | Abroad | n.a.* | n.a.* | n.a.* | n.a.* |
|  | Abroad | Netherlands | n.a.* | n.a.* | n.a.* | n.a.* |
|  | **Abroad** | Abroad | n.a.* | n.a.* | n.a.* | n.a.* |
| *third generation* | Netherlands | Netherlands | **Abroad** | Abroad | Abroad | Abroad |
|  | Netherlands | Netherlands | Abroad | Abroad | Netherlands | Netherlands |
|  | Netherlands | Netherlands | Netherlands | Netherlands | Abroad | Abroad |
|  | Netherlands | Netherlands | **Abroad** | Abroad | Abroad | Netherlands |
|  | Netherlands | Netherlands | Abroad | Abroad | Netherlands | Abroad |
|  | Netherlands | Netherlands | Netherlands | Abroad | **Abroad** | Abroad |
|  | Netherlands | Netherlands | Abroad | Netherlands | **Abroad** | Abroad |

**bold**: in case of different birth countries, the bold one decides on child’s ethnicity.

* At least one of the grandparents is born abroad.
