# Supplementary material for: The role of early life factors in the development of ethnic differences in growth and overweight in preschool children: a prospective birth cohort
Source: BMC Public Health. 2014 Jul 15;14:722. doi: 10.1186/1471-2458-14-722 (PMC4227130; doi:10.1186/1471-2458-14-722)
Supplement: Additional file 2 — Main results (Table 2) for unimputed data (available case analysis). [file 1471-2458-14-722-S2.docx]

Appendix 2: Associations between ethnicity and overweight at 48 months, and contribution of early life factors in the Generation R Study – not imputed estimates^a^

|  | Model 1  (n=2994) | Model 2  (n=2259) | Model 3  (n=1729) | Model 4  (n=2994) | Model 5  (n=1489) | Model 6  (n=808) |
| --- | --- | --- | --- | --- | --- | --- |
| Child’s ethnicity |  |  |  |  |  |  |
| *native Dutch* | 1 (ref) | 1 (ref) | 1 (ref) | 1 (ref) | 1 (ref) | 1 (ref) |
| *Turkish* | **3.25 (2.39-4.41)** | **2.08 (1.30, 3.31)** | **2.07 (1.26-3.39)** | **3.69 (2.69-5.06)** | **2.30 (1.42-3.73)** | 2.05 (0.72-5.82) |
| *Moroccan* | **2.40 (1.66-3.47)** | **1.79 (1.01, 3.14)** | 1.43 (0.71-2.89) | **2.63 (1.81-3.83)** | 1.92 (0.99-3.71) | 0.97 (0.19-4.89) |
| *Cape Verdean* | **2.00 (1.22-3.27)** | 1.64 (0.88, 3.05) | 1.46 (0.64-3.36) | **2.63 (1.59-4.37)** | 0.81 (0.29-2.33) | 0.88 (0.15-5.15) |
| *Antillean/Surinamese-Creole* | **1.82 (1.16-2.87)** | 1.23 (0.63, 2.40) | 0.71 (0.25-2.03) | **2.25 (1.41-3.57)** | 0.99 (0.41-2.38) | 0.94 (0.11-7.81) |
| *Surinamese-Hindu* | 0.89 (0.41-1.96) | 0.96 (0.40, 2.33) | 0.82 (0.27-2.44) | 1.39 (0.62-3.09) | 0.67 (0.16-2.88) | 0.78 (0.06-9.49) |
| Household income |  |  |  |  |  |  |
| *low* |  | 0.87 (0.58-1.31) |  |  |  | 1.09 (0.44-2.72) |
| *above average* |  | 1 (ref) |  |  |  | 1 (ref) |
| Mother’s educational level |  |  |  |  |  |  |
| *high* |  | **0.50 (0.31-0.81)** |  |  |  | 0.65 (0.19-2.24) |
| *middle* |  | **0.40 (0.23-0.69)** |  |  |  | 0.67 (0.18-2.42) |
| *low* |  | 1 (ref) |  |  |  | 1 (ref) |
| Material hardship |  |  |  |  |  |  |
| *no* |  | 1 (ref) |  |  |  | 1 (ref) |
| *yes* |  | 1.35 (0.93-1.96) |  |  |  | 1.21 (0.53-2.79) |
| Mother’s BMI (per unit) |  |  | **1.09 (1.06-1.13)** |  |  | 1.05 (0.99-1.11) |
| Father’s BMI (per unit) |  |  | **1.15 (1.10-1.20)** |  |  | **1.20 (1.11-1.30)** |
| Smoking during pregnancy |  |  |  |  |  |  |
| *no* |  |  | 1 (ref) |  |  | 1 (ref) |
| *yes* |  |  | 1.53 (0.95-2.48) |  |  | **2.71 (1.22-5.99)** |
| Birth weight (per 500 gram) |  |  |  | **1.05 (1.04-1.07)** |  | **1.08 (1.05-1.12)** |
| Gestational age (per week) |  |  |  | **0.85 (0.78-0.93)** |  | 1.00 (0.80-1.25) |
| Breastfeeding at 6 months |  |  |  |  |  |  |
| *no* |  |  |  |  | 1 (ref) | 1 (ref) |
| *yes* |  |  |  |  | 1.31 (0.91-1.88) | 0.91 (0.51-1.60) |
| Difference in BMI between birth and 6 months (per unit) |  |  |  |  | **1.31 (1.13-1.53)** | **1.52 (1.18 -1.97)** |

^a^ Values are odds ratios (95% confidence intervals)

Model 1: adjusted for age and sex

Model 2: adjusted for sex, age, and socio-demographics (household income, mother's education, material hardship)

Model 3: adjusted for sex, age, and parental (parental BMI, maternal smoking during pregnancy)

Model 4: adjusted for sex, age, and birth characteristics (birth weight and gestational age)

Model 5: adjusted for sex, age, and postnatal factors (breastfeeding, infant weight gain)

Model 6: adjusted for all variables in model 1-5 simultaneously
